# Production of Transgenic Pigs Mediated by Pseudotyped Lentivirus and Sperm

**DOI:** 10.1371/journal.pone.0035335

**Published:** 2012-04-20

**Authors:** Yongliang Zhang, Qianyun Xi, Jinghua Ding, Weiguang Cai, Fanmin Meng, Junyun Zhou, Hongyi Li, Qingyan Jiang, Gang Shu, Songbo Wang, Xiaotong Zhu, Ping Gao, Zhenfang Wu

**Affiliations:** Guangdong Provincial Key Laboratory of Agro-Animal Genomics and Molecular Breeding, College of Animal Science, South China Agricultural University, Guangzhou, China; University of Connecticut, United States of America

## Abstract

Sperm-mediated gene transfer can be a very efficient method to produce transgenic pigs, however, the results from different laboratories had not been widely repeated. Genomic integration of transgene by injection of pseudotyped lentivirus to the perivitelline space has been proved to be a reliable route to generate transgenic animals. To test whether transgene in the lentivirus can be delivered by sperm, we studied incubation of pseudotyped lentiviruses and sperm before insemination. After incubation with pig spermatozoa, 62±3 lentiviral particles were detected per 100 sperm cells using quantitative real-time RT-PCR. The association of lentivirus with sperm was further confirmed by electron microscopy. The sperm incubated with lentiviral particles were artificially inseminated into pigs. Of the 59 piglets born from inseminated 5 sows, 6 piglets (10.17%) carried the transgene based on the PCR identification. Foreign gene and EGFP was successfully detected in ear tissue biopsies from two PCR-positive pigs, revealed via in situ hybridization and immunohistochemistry. Offspring of one PCR-positive boar with normal sows showed PCR-positive. Two PCR-positive founders and offsprings of PCR-positive boar were further identified by Southern-blot analysis, out of which the two founders and two offsprings were positive in Southern blotting, strongly indicating integration of foreign gene into genome. The results indicate that incubation of sperm with pseudotyped lentiviruses can incorporated with sperm-mediated gene transfer to produce transgenic pigs with improved efficiency.

## Introduction

In 1989, two independent reports claimed that sperm cells could associate with exogenous DNA molecules and transfer of these molecules during fertilization, resulting in genetically modified offspring [1], [2]. This process, termed sperm-mediated gene transfer (SMGT), provides a simple and straightforward method to produce transgenic animals. However, the SMGT protocol has been extended to many animal species, including mice [3], [4], rats [5], rabbits [6], and pigs [7], [8]. It has also been a controversial issue in the past two decades as several other groups reported failure in repeat the original gene transfer protocol [9]. The underlying causes for the laboratory-to-laboratory and species-to-species variations observed by different researchers are still not clear. It was believed that the generation of non-integrated episomal structures is a highly probable event [10], [11], rare integration was observed [12], [13]. The foreign DNA would be transmitted to the next generation by being maintained as an extrachromosomal structure (episome) in the positive transgenic animals [14], [15]. This may account for non-stable inheritance in SMGT with plasmid DNA [8], [16], [17]. Therefore, SMGT has not been widely adopted for making transgenic animals.

Recently, lentiviral vectors have been proved to be superior to plasmid DNA in production of transgenic animals. The benefit in using the lentiviral vector system is its capability to efficiently integrate into the host genome [18]. Integration of lentiviral vectors is less random than plasmid DNA, and prefers active transcription units [19], [20]. It was found that lentiviral vectors based on HIV-1 integrated within transcriptional units [21]. There were 1–5 or higher integrants in transgenic pigs by using lentiviral vectors. Furthermore, pigs have exhibited transgenic rates of 70–93% with a lentivirus system [22]. Lentiviral vectors have become an appealing tool for transgenesis because of their abilities to incorporate transgene into genomic DNA with high efficiency. In addition, the transgene expression by lentiviral vector can also be maintained. The embryo viabilities following lentiviral vector transduction has been shown to be very high (generally>70%) in many animal species, including mice [23], [24], rats [23], pigs and cows [22], [25]. By injection of lentiviral vector carrying the green fluorescent protein (GFP) into the perivitelline space, of the 46 piglets born, 32 (70%) carried the transgene DNA and 30 (94%) of these pigs expressed the transgene [22]. The lentiviral vectors are capable of transducing mouse and rat spermatogonial stem cells (SSCs) [26], [27], in which transgenic offspring were produced after transplantation. Hamra et al. [27] reported that rat germ-line cells were transduced with a lentiviral enhanced GFP reporter vector and then transferred to WT recipient males, after mating, 13 pups carried the lentiviral transgene.

Most studies have been used injection of the lentivirus into the perivitelline space of fertilized oocytes or early embryos, followed by surgical implantation to recipient animals [23]. No studies have examined infection of lentiviral vectors to sperm cells, and then used the infected sperms as carrier to inseminated oocyte for transgene delivery. Conceivably, it is likely that incubation of lentiviral vectors with sperm would be an even more effective and economical method than virus injection to perivitelline space. Therefore, we explored the development of a simple way to produce transgenic pigs by incubation of sperm with lentivirus.

## Results

### Association of lentiviruses with pig spermatozoa

**Figure 1:**
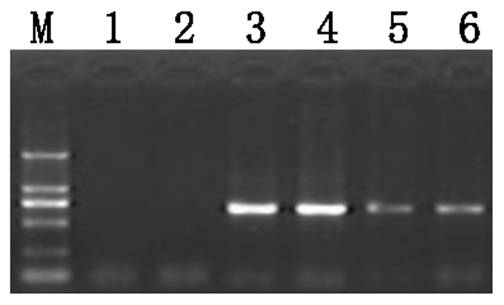
RT-PCR amplification of a specific fragment of the lentiviral vector after incubation with sperm cells. Lane M: DNA Ladder DL2000 (2000, 1000, 750, 500, 250 and 100 bp from top to bottom). Lane 1: Negative control with H_2_O as template. Lane 2: Negative control with total RNA extracted from sperm alone as template, both of which showed no PCR products. Lanes 3–6: Specific PCR products were detected in sperm samples incubated with lentiviral particles for 2, 4, 6 and 8 h, respectively.

**Figure 2:**
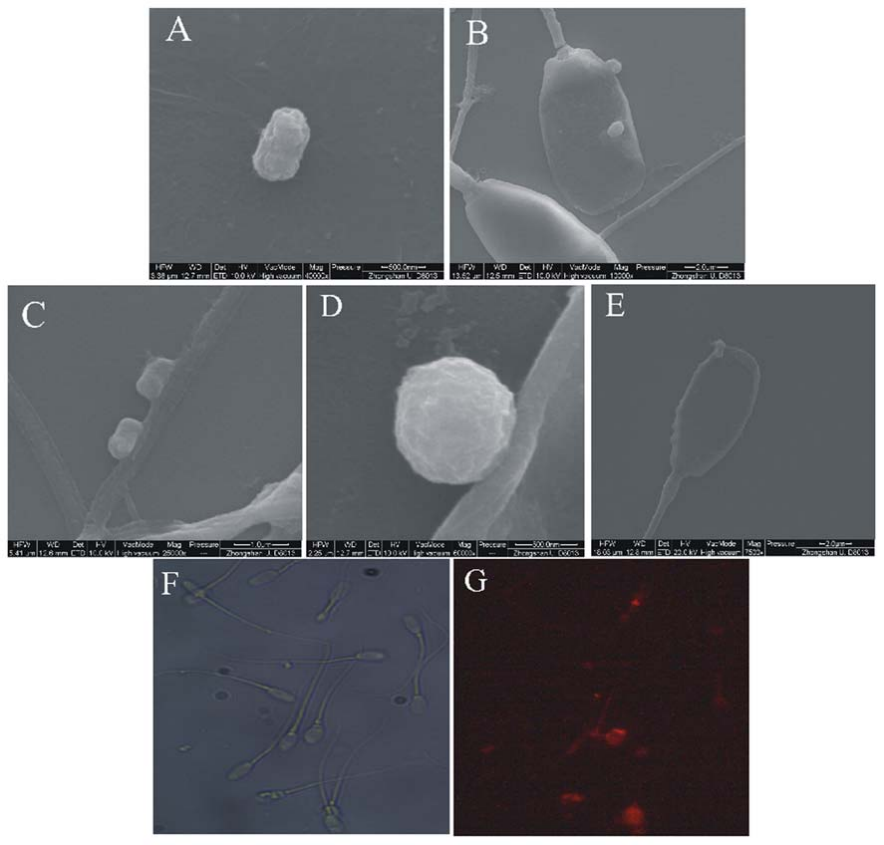
Absorption of lentiviral particles by pig spermatozoa under a scanning electronic microscope and immunochemical detection of lentiviral particles on spermatozoa. A: lentiviral particles (40000×). B: Two lentiviral particles are clearly attached on the head of one spermatozoa (10000×). C: Two lentiviral particles absorbed on a spermatozoa tail (25000×). D: Observation of interaction between a lentiviral particle and the spermatozoa surface in an enlarged image (60000×). E: A lentiviral particle entering a spermatozoon (7500×). Immunuchemical detection of lentiviral particles attached on sperm was performed using a monoclonal mouse anti-VSV-Glycoprotein antibody, F was view field of porcine spermatozoa (200×), and red fluorescence was clearly observed emitted at 570 nm (G, 200×).

To determine whether pig spermatozoa could associate with the lentiviruses, we simply incubated pig spermatozoa with exogenous lentiviruses. By RT-PCR amplification of the RNA specific sequence of the lentivirus absorbed by sperm, the results showed that the lentiviral mRNA was present in the incubated sperm after several times of washing (Figure 1). Moreover, qRT-PCR was performed to quantify mRNA of the lentiviral particles after spermatozoa and virus incubation. Ct value was plotted against known lentiviral number to draw the standard curve (y = −0.207*Log (x)+27.04) (Figure S1), Lentiviral particles was calculated with Ct value. The results showed that one hundred spermatozoa absorbed approximately sixty-two lentiviral particles after incubation for 4 h (Table 1). The lentiviral mRNA level associated with the transgene construct in the incubated sperm did not increase with longer incubation periods. The number of lentiviral particles taken up by spermatozoa significantly decreased (P<0.01) after incubation for 6 h and 8 h compared with 4 h incubation. The association of pig spermatozoa with lentiviral particles was further confirmed by direct observation under field scanning electronic microscope (Figure 2). The images of incubated sperm clearly illustrated that lentiviral particles were attached on the spermatozoa heads and tails. Lentiviral particle was observed in the stage of crossing the sperm membrane in some sperms.

**Table 1:** Number of lentiviral particles associated with sperm cells after incubation (n = 3).

Incubation time	2 h	4 h	6 h	8 h
absorbed lentiviral particles/100 spermatozoa	15±2	62±3	35±4	34±2

Immunuchemical detection of lentiviral particles attached on sperm was performed using a monoclonal mouse anti-VSV-Glycoprotein antibody. Red fluorescence was clearly observed on some sperms (Figure 2, F, G), which verified the lentiviral particles attached on the surface of pig spermatozoa.

### Generation of transgenic pigs by insemination with sperm incubated with lentiviruses

Sperm motility was calculated to be 0.8 before and 0.7 after incubation with lentivirus. Six sows were inseminated with sperm incubated with lentiviruses. Five sows became pregnant and one had a spontaneous abortion; the remaining four sows produced 59 piglets (Table 2). Six piglets (3 males, 3 females) of the 59 piglet born from the five sows (10.17%) were positive in the PCR amplification of the specific 649 bp fragment, which joins the woodchuck hepatitis virus response element (WPRE) and EGFP in a lentivirus vector. The genomic DNA for the PCR amplification was isolated from 3 d old pig ear biopsies. The 649 bp foreign gene fragments were detectable at day 60, 120, 180 and 270 while the age-matched control piglets were negative (Figure S2). Semen samples from three male pigs were collected, and spermtozoa DNA was examined via PCR amplification, and all three DNA samples were positive (Figure S3). The results of PCR from organs and tissues in one piglet, such as heart, liver, spleen, kidney, lung, stomach, brain, ovary, cerebral cortex, belly fat, semitendinosus muscle, semimembransus muscle, longissimus dorsi muscle and duodenum, were all PCR-positive (Figure S4). These results imply that the transgene delivered by the lentiviral vector might be transmitted to the next generation.

**Table 2:** PCR amplification of transgene DNA sequences in piglets.

Lentiviruses (5×10^5^ ifu/ml, ml)	Number of sperm incubated with lentiviruses	Source of DNA template for PCR amplification			
		Ear biopsy from 3 day old piglet		Ear biopsy from 30 day old piglet	Ear biopsy from 120 day old piglet
		No. of piglets	No. of PCR-positive piglets (%)	No. of PCR-positive piglets	No. of PCR-positive piglets
10	1×10^9^	59	6 (10.17%)	6	6

The expression of EGFP was not observed by direct epifluorescence of the body surface. Further analysis of EGFP expression after sacrifice of a 230-day transgenic pig indicated the existence of the transgene in this pig. The RT-PCR results revealed the presence of the transgene EGFP mRNA in the kidney and ovary, and weak expression in the heart and lungs (Figure S5A). Direct detection of EGFP was observed in the kidney and ovary under ultraviolet light at 380 nm wavelength (Figure S5B and S5C). This results further confirmed transgene expression in the transgenic piglet.

**Figure 3:**
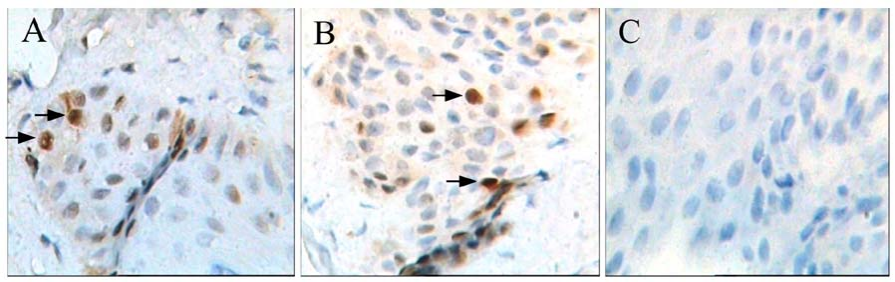
In situ hybridization detecting EGFP gene in PCR-positive transgenic pigs. Most ear cell nucleus from two pigs were stained to be brown. A (400×) was for pig No. 18 and B (400×) was for No. 41. C (400×) was control sample from normal pig ear with negative staining. All nucleus were stained to be blue.

In situ hybridization and immunohistochemistry were used to detect GFP gene and GFP in ear tissue biopsies from two PCR-positive pigs. In slides from two pigs, most of the cells were positive (in brown) in nuclei, revealed via in situ hybridization (Figure 3A and 3B). And most of the cells also showed brown in cytoplasm, revealed via immunohistochemistry (Figure S6A and S6B). Results above strongly support the idea that EGFP DNA had integrated into pig genome, and was expressed successfully in cells.

Ten piglets were produced from two sows after artificial insemination using semen from one PCR-positive boar. Interestingly, PCR amplification of transgene (649 bp) of piglets was all positive (Figure S7).

**Figure 4:**
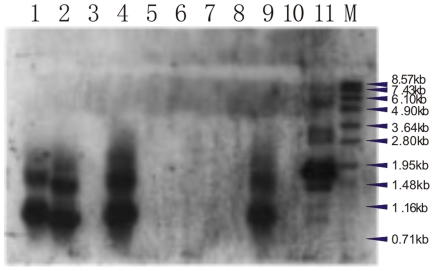
Identification of transgenic pigs by Southern blot analysis. Southern blot was performed under optimized condition with a Dig-lableled probe of 649 bp fragment. Samples in lanes 1 and 2 are from PCR-positive the founder No. 18 and No. 41, respectively, lanes 3–8 are their offsprings; The specific hybridization signal was detected in Lanes 1, 2, 4, 9; lanes 3, 5, 6, 7, 8 are negative, respectively; lane 10 is wild type control; lane 11 is positive controls (The plasmid pLV-siRNA); M is DNA Molecular Weight VII (DIG-labeled Roche).

Two PCR-positive founders and offsprings of PCR-positive boar were further identified by Southern-blot analysis (Figure 4), out of which the two founders and two of the seven offsprings (28.57%) were positive in both Southern blotting and PCR, others were positive only in PCR. All southern-positive transgenic pigs showed the presence of one to three bands that hybridized with the 649 bp fragment probe, indicating one to three copies of the transgene integrated into the genome of the transgenic hosts. Unlike this, wild type control (WT) did not show the specific hybridization signal. Southern blot hybridizations further supported the results of PCR, RT-PCR, in situ hybridization and immunohistochemistry analysis and confirmed that the copy number of integration into genome was low. These results provided strongly evidence for the integration of foreign gene into pig genome.

## Discussion

Production of transgenic farm animals have been challenged by low efficiency, high cost of time and labor due to the difficulty in manipulation of embryos at early stages of development [28]. Lentiviral gene transfer in fertilized oocytes and embryo improved the efficiency significantly, but it still needs expensive micromanipulators. In this research, we described at the first time the production of transgenic pigs by using sperm incubated with pseudotyped lentiviral particles, offering a simple and cost-effective method for generating transgenic animals although the efficiency and level of transgene expression are still low and needs further experiments.

It is believed that spermatozoa of virtually all animal species have the spontaneous ability to take up exogenous DNA molecules and to deliver them to oocytes at fertilization. Research results also showed that RNA molecules were captured by sperm cells [15], [29], exogenous RNA may be internalized in spermatozoa via a membrane receptor, and then was reverse-transcribed to cDNA by reverse transcriptase in spermatozoa, and the retro-genes were delivered to oocytes and transmitted to embryos and offspring with low copy numbers. It remains unclear how lentiviral particles attach or are absorbed on sperm surface, or if they are internalized into the sperm cell and integrated in the genome. In fact, it has been reported that HIV-1 was detected in human spermatozoa by PCR [30], and HIV-1 may be harbored on the human sperm tail [31]. However, the peudotyped lentivirus utilized in our study contains a VSV-G envelope, and it is not clear how these surface proteins interact with the pig spermatozoa. It is unclear how effective lentiviral particle can bind spermatozoa in livestock animals. The results from this study revealed that pig spermatozoa could associate with lentiviral particles. Lentiviral fragments were detected by RT-PCR in washed pig spermatozoa after incubation with lentiviruses. Pig spermatozoa were able to absorb lentiviral particles at a maximum level after 4 h incubation. The direct observation under an electronic microscope showed that lentiviral particles were attached on the surface of spermatozoa heads or tails, which was further proved with immunochemical detection of lentiviral particles on spermatozoa using VSV-G antibody. The nature of the interaction between lentiviral particles and the surface of the pig spermatozoa remains unclear. This association may be non-specific or specific to certain molecules on the spermatozoa membrane. Further experiments with in vitro sperm incubation may reveal the underlying mechanism of virus entry and subsequent gene transfer, which may provide important information for understanding the detailed interactions between lentiviral particles and spermatozoa.

In this study, we showed that pig spermatozoa were able to carry the lentivirus to the oocyte and thereafter produced transgenic pigs. The efficiency of producing a transgenic pig by using lentivirus incubation with sperm was 10.17%. The titer of the unconcentrated lentivirus used was 5×10^5^ ifu/ml. It may be possible to achieve a higher efficiency of transgenesis if a high titer of lentivirus was used, but toxicity of lentivirus to sperm may be also increased. This point needs further exploration. In one piglet, the foreign gene was detected in all tested organs and tissues, and EGFP mRNA was detected by qRT-PCR in kidney, ovary, heart and lung. In situ hybridization revealed that foreign gene had most possibly integrated into PCR-positive pig genome, and EGFP was also detectable in ear cells in immunohistochemical detection. But EGFP fluorescence was not observed on pig ear, probably due to lower expression level. Sperm from three transgenic pigs were also positive for the transgene, suggesting that the foreign gene might be transmitted to the next generation. Ten offsprings from one PCR-positive male pig showed all PCR positive.

PCR assay can be commonly used to screen the positive samples first, and Southern blotting can be used with the reduced sample size [32]. Therefore, all PCR-positive pigs were further screened by southern blotting. As a result, four southern-positive pigs (two founders and two offsprings) were detected (Figure 4). These results strongly indicated that the foreign gene was integrated into the host genome, and transmitted from founder to the offspring via the sperm. But the result of southern blotting also showed most of the transgenic events had low copy number (1–3 copies), which was lower than the 1–20 copies (mean = 4.6) with standard pronuclear microinjection method reported by Hofmann et al. [33]. At the same time, some of PCR positive offsprings had no hybridization signal in the southern blot analysis (southern-positive rate = 28.57%). The discrepancy in the results of PCR and Southern blot hybridization analysis seemed to reflect their sensitivity. PCR quite often produces false positive data due to the low primers specificity. Compared to the high sensitivity of PCR test for detecting target gene, Southern blotting has a lower sensitivity, but it also has a high specificity which is important to reduce false positive results and rate of contamination [34].

All transgenic pigs produced in this work did not show any health problems, so far as observed. Although all organs and tissues from this piglet with EGFP protein expression were positive by PCR detection, EGFP was not detected on the body surface skin. Several studies [23], [35], [36] have indicated that genetic mosaicism occurs using lentiviral vectors. May et al. [37] reported that lentivirus vectors could be subjected to gene silencing, and epigenetic modifications for gene expression. The site of proviral integration could markedly affect the level of GFP expression [38], and that the effects on transgene expression may be associated with a phenomenon known as position effect variegation in which integration within particular chromosomal regions results in altered transgene expression [39]. The integration site may inevitably influence the expression of EGFP transgenes in pigs. Furth et al. [40] reported that levels of a reporter gene expression under the control of a CMV promoter (CMVp) varied dramatically among tissues, and the highest levels were in the heart, stomach and spleen. CMVp is not a universal promoter in vivo, and gene silencing has occurred when CMVp was used to produce transgenic animals [40], [41]. While the DNA samples from ear tissue of transgenic pigs were all positive during PCR amplification in our study, the EGFP was not observed on body surfaces by direct fluorescence imaging. There remains little doubt that further work on variable gene expression and silencing effects will be needed to validate the use of specific promoters, and that specific cellular promoters will likely prove to be superior over general viral promoters for lentiviral transgenesis.

### Conclusions

The main target of this study was to establish new methodology of transgenic delivery. Due to the method of microinjection requires a high condition of technology (cell culture, microinjection, embryo manipulation, transplantation, etc) and equipment, thus its extensive application has been significantly limited. In this study, although compared with microinjection of lentivirus and transposon, the efficiency of producing a transgenic pig by using lentivirus incubation with sperm was lower, this transgenic approach is surely a maneuverable, low-tech and low-cost method of transgene delivery. While a higher efficiency for transgenesis achieved by this method needs further exploration.

## Materials and Methods

### Animals

The experiments were carried out in a pig-breeding farm located in the southern region (Yunfu City) of Guangdong province in China. Pig semen was obtained from one Landrace boars and 6 Landrace sows were inseminated by the semen. The animal care and use protocol for this study was approved by the University's Institutional Animal Care and Use Committee, all pigs were treated humanely.

### Ethics Statement

All of the animal slaughter experiments were conducted in accordance with the guidelines of Guangdong Province on the Review of Welfare and Ethics of Laboratory Animals approved by the Guangdong Province Administration Office of Laboratory Animals (GPAOLA). All animal procedures were conducted under the protolcol (SCAU-AEC-2009-0326) approved by the Animal Ethics Committee of South China Agricultural University.

### Pseudotyped lentivirus

The lentiviral vectors (four plasmid system, SBI, USA) were extensively modified to carry a cytomegalovirus (CMV) promoter driving expression of enhanced green fluorescent protein (EGFP) and the histone 1 (H1) promoter downstream to allow the efficient introduction of oligonucleotides encoding shRNA. There was not any shRNA gene included in the vetor.

### Generation of lentivirus

**Figure 5:**
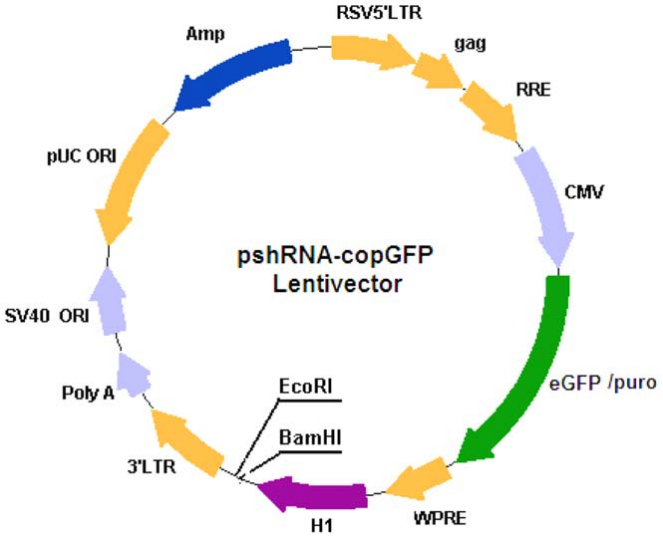
Map of lentiviral vector.

The lentiviral vectors included four plasmid, pSHR-Puro/GFP, pPACK-GAG, pPACK-REV and pVSV-G (SBI, Los Angel, USA), SHR-Puro/GFP carried a cytomegalovirus (CMV) promoter driving expression of enhanced green fluorescent protein (EGFP) and the histone 1 (H1) promoter downstream to allow the efficient introduction of oligonucleotides encoding shRNA. There was not any shRNA gene included in the vector. Lentiviral production was performed as described [42]. Briefly, pshRNA-copGFP Lentivector Vector (LV-shRNA-GFP) (Figure 5) and three packaging vectors, pPack A (pREV), pPack B (pVsv-g) and pPack C (pGag-pol) were co-transfected into 293T cells and the resulting supernatant was collected after 48 h. The supernatant was cleared of cell debris by filtering through a 0.45 µm filter, and the titer of the lentivirus in the supernatant was determined (5×10^5^ infections units (ifu)/ml) by using cell dilution method according to manufacture's guide.

### Incubation of sperm with lentivirus and artificial insemination

Semen was collected from a Landrace boar. Sperm density is determined by SEMCHECK 2 COLORIMETER (Rotech Livestock Equipment Ltd, UK.). Sperm was washed to remove seminal fluid [8]. After three steps of washing with 40 ml SFM/BSA medium followed by 800 g centrifuge for 10 min, collected sperms were incubated in SFM/BSA solution, which is made of 11.25 g anhydrous glucose, 10 g sodium citrate dihydrate, 4.7 g dihydrate disodium ethylenediamine tetraacetic acid monohydrate, 3.25 g citric acid monohydrate, and 6.5 g trishydroxymethyl aminomethane in 1 liter of double distilled water, with 6 g bovine serum albumin added before use. Initially, 5 ml of fresh semen were cultured for 5 min in the presence of 5 ml SFM/BSA medium at 37°C. Then the solution was transferred to a preheated (25°C) 50 ml centrifuge tube containing 40 ml SFM/BSA. After centrifugation at 800 g for 10 min, the spermatozoa pellet were gently re-suspended in a 50 ml centrifuge tube with 40 ml 25°C-preheated SFM/BSA solution. The resuspended solution was centrifuged for 10 min at 800 g at 17°C. After complete and careful discard of the supernatant, the sperm were gently resuspended in 3∼4 ml preheated (17°C) SFM/BSA. Washed sperm (1×10^9^ for each artificial insemination) were incubated with 10 ml lentivirus supernatant (5×10^5^ ifu/ml) at 17°C in 40 ml SFM/BSA for 2 h. The sperm solution flask was inverted every 20 min to prevent sedimentation of the sperm. For the final 20 min of incubation of the 2 h, the sample was kept at room temperature. Sperm motility was calculated before and after incubation with lentivirus using red blood count under microscope. Just before artificial insemination, the sample was heated at 37°C for 1 min. Sperms (1×10^9^) in 40 ml SFM/BSA were inseminated into uterine of Landrace sow. Six Landrace sows were received two inseminations and both were done according to the manner described.

### Evaluation of association of pseudotyped lentivirus with sperm *in vitro*

Quantitative RT real-time PCR (qRT-PCR) was used to examine the association of pseudotyped lentiviruses with sperm, and to calculate the lentiviral particle number present in sperm. Washed sperm (1×10^9^) cells were incubated with 10 ml lentivirus supernatant (5×10^5^ ifu/ml) at 17°C in 40 ml SFM/BSA. After incubation for 2, 4, 6 or 8 h, the triplicate sperm samples (2 ml each sample) were taken at each time point and washed with 10 ml PBS 3 times. RNA was extracted from the washed sperm by QIAamp Viral RNA mini kit, according to manufacturer's instructions (Qiagen, Valencia, CA, USA), eluted in 60 µl of elution buffer. RNA samples (1 µl elution) was incubated at 65°C for 10 min with 1 µl (20 µM) oligodT18 primers. The samples were then cooled on ice for 2 min and briefly centrifuged. The following components were added to the mRNA/primer solution: 4 µl 10× AMV buffer, 1.0 µl RNase Inhibitor (40 U, Takara, Dalian, China), 1 µl dNTPs 2.5 mM), 0.5 µl AMV (0.25 U, Takara, Dalian, China), and RNase-free water to attain a total volume of 20 µl; The set-up was then incubated at 37°C for 1.5 h. The reaction mixture was then heated to 95°C for 10 min to heat-inactivate the reverse transcriptase enzyme. Quantitative real-time PCR was performed in triplicate with SYBR Green in an Agilent Stratagene Mx3005P Sequence Detection (La Jolla, CA, USA). The sequences of the primers targeting 137 bp sequence in EGFP were: forward 5′-CTACGGCTTCTACCACTTCG-3′; and reverse 5′-CGTCCTCGTACTTCTCGATG-3′. Standard curves were generated by using 10-fold serial dilutions (1×10^5^ copies to 10 copies/10 µl) of lentivirus (dotting lentiviral particle number against Ct value). The number of lentiviral particles associated with spermatozoa was calculated against the standard curve.

Absorption of lentivirus by sperm was also observed under field emission scanning electron microscopy (FESEM, Jeol JSM-6330F) after 4 h of incubation as described above. The sperm absorbed lentivirus were centrifuged at 500 g for 10 min and fixed for 2 h in 2.5% glutaraldehyde in 0.1 mol/L cacodylate buffer (pH 7.4). After washing with PBS (pH 7.4), the sperm were post-fixed in 2% osmium tetroxide plus 1% potassium ferricyanide in cacodylate buffer, dehydrated through a graded alcohol series, freeze-dried, and conductively coated with platinum. Samples were examined uncoated with the field emission scanning electron microscopy (FESEM) at low accelerating voltage (5 kV) in the Electronic Microscope Center in Sun Yat-Sen University (Guangzhou, China).

Incubation of pig sperm and lentiviral particles was as same as described before. For the detection of VSV glycoprtein of lentiviral particles on spermatoaoa, we applied the immunofluorescence assay. Briefly, the detection was performed on U-bottomed 6-well assay plates pre-treated with poly-L-lysine hydrobromide in order to increase their adhesive capacity. The plates were loaded with 1 ml/well washed sperm suspension after incubation with lentivirus, and then were methanol fixed for 30 min. The supernatant was removed and washed by PBS. Non-specific binding was reduced by incubating with bovine blocking buffer: 1% bovine serum albumin (BSA) in PBS. The incubates with a monoclonal mouse anti-VSV-Glycoprotein antibody labeled with Cy3 (1∶1000 dilution, protein clone P5D4, C-7706; Sigma-Aldrich, St. Louis, MO, USA) for 15 min was done after all the supernatant discarded. The supernatant was removed and washed by PBS as described previously. Results were observed using a fluorescence microscope (DMIL, Leica, Germany) emitted at 570 nm.

### Identification of transgenic pigs using PCR amplification of foreign gene

Ear tissue biopsies were collected from 59 piglets born from the inseminated sows on postnatal day 3, 60, 120, 180 and 270. Genomic DNA was isolated from the ear tissue by proteinase digestion, followed by phenol and chloroform extractions. Transgene DNA was detected by PCR amplification of a 649 bp fragment linking the lentiviral and EGFP DNA sequences. The primer sequences were: 5′-CTACGGCTTCTACCACTTCG-3′ (forward); and 5′-GCAGCGTATCCACATAGCGT-3′ (reverse). The conditions for PCR were as follows: 2.5 µl 10× PCR buffer, 0.2 mM dNTPs, 0.4 mM each primer, 2.5 U Taq polymerase (Takara, Dalian, China), 1 µl (0.5 µg) DNA, and 17 µl sterile filtered water; the final volume was 25 µl. The PCR reaction was carried out by established conditions (35 cycles of 94°C for 30 s, 60°C for 30 s, and 72°C for 30 s). Resulting PCR products were electrophoresed on a 1.5% agarose gel. The plasmid DNA of pLV-siRNA and wild-type pig genomic DNA were used as positive and negative controls in the PCR amplification. The amplified fragments were sequenced using an ABI PRISM 377 DNA sequencer (PerkinElmer, Massachusetts, USA).

One PCR positive piglet was sacrificed at 230 days old, and its kidney, heart, lung, liver, spleen, and ovary were dissected and sampled. Enhanced green florescent protein fluorescence were observed under MLS macroscope (Biological laboratory equipment maintenance and service. ltd). Total RNA was also extracted from the organs and tissues and 1 µg of total RNA was used as template for reverse transcription with oligo dT (13) as the primer. Reverse transcriptase-PCR reactions included 6 µl cDNA and primers in transgene detection described above and were carried out according to the same procedure. RNA templet from normal pig was taken as negative control, the amplified fragment was 137 bp. Resulting PCR products were electrophoresed on a 1.5% agarose gel.

Semen samples were collected from three PCR-positive male pigs (210 days of age). Genomic DNA was extracted for PCR analysis described above. The plasmid pLV-siRNA was used as positive controls and normal pig ear DNA and water as negative controls.

### Identification of transgenic pigs using in situ hybridization and immunohistochemisty detecting EGFP gene and its expression

In situ hybridization and immunohistochemistry to detect EGFP gene and its experession were conducted in Jingyang Company (Tianjin, China). Ear tissue biopsies were collected from 2 remaining PCR positive pig (No. 18 and No. 41, age 54 monthes). Ear samples were fixed in 4% formaldehyde overnight, washed in 70% ethanol, dehydrated in graded series of alcohols, and embedded in Paraffin. Serial and adjacent sections, 7–8 µm thick, were cut in sagittal planes and mounted on glass slides.

For in situ hybridization, glass slides had been pretreated with 3-aminopropyltriethoxysilane, allowed to dry, and baked overnight at 65°C. The sections were then deparaffinized with xylene and alcohol and again allowed to dry. Sections were placed in Protein Digesting Enzyme solution and incubated at 37°C for 15 minutes. Following digestion, the sections were washed with PBS and treated with 0.1% hydrogen peroxide in PBS for 30 min to quench endogenous peroxidase activity. Hybridization was performed as recommended by the manufacturer of the biotin-labeled probe targeting EGFP (GCGTT GCTGC GGATG ATCTT GTCGG TGA, Jingyang, Tianjin, China). Hybridization was then amplified by the addition of Streptavidin-HR (Jingyang, Tianjin, China) diluted 1∶50 in PBS. Tissue sections were washed in PBS, followed by incubation in 0.05% 3, 3-diaminobenzidine hydrochloride (DAB) containing 0.02% hydrogen peroxide for 7 min. Following washes in PBS, sections were incubated by hematoxylin for 1∼3 min. After washing with PBS, sections were incubated by 1% hydrochloric acid solution for 10 s. Sections were washed, dehydrated and cleared in increasing concentrations of ethanol and finally xylene, coverslipped with neutral balsam. Finally, nuclei were stained with hematoxylin and the sections were viewed with a optical microscope (OlympusSZX7, Japan).

For immunohistochemistry, the paraffin sections were baked, dewaxed, hydrated, rinsed in PBS for 10 min and treated with 0.1% hydrogen peroxide in TBS for 30 min to quench endogenous peroxidase activity. Slides were first incubated with heat-inactivated 5% normal goat serum in PBS at pH 7.6, for 30 min at room temperature and then incubated at 4°C for 60 min in Rabbit polyclonal anti-CopEGFP antibody (1∶50, AB501, Evrogen, Russia) diluted in PBS. Slides were washed and incubated with goat anti-rabbit IgG antiserum (Jingyang, Tianjin, China) diluted 1∶25 in PBS with 5% goat serum for 1 h at 37°C. After another 20 min PBS wash, the sections were incubated for 30 min in Streptavidin-HRP (Jingyang, Tianjin, China) diluted 1∶50 in PBS. The following procedure was exactly the same as described in in situ hybridization.

### PCR detection of offspring of one PCR-positive male pig

Semen were collected from one PCR-positive male pig and inseminated artificially to two normal female pigs as described before. Ear tissue biopsies were collected from 10 newborn piglets, genome DNA was isolated and transgene DNA was detected by PCR amplification, as described before. Resulting PCR products were electrophoresed on a 1.5% agarose gel, and sequenced using an ABI PRISM 377 DNA sequencer (PerkinElmer, Massachusetts, USA).

### Identification of transgenic pigs by Southern blot analysis

Genomic DNA from ear tissues of positive transgenic pigs were extracted as described above, the plasmid pLV-siRNA was used as positive controls and wild type pig ear DNA as negative controls. For Southern blot analysis of transgenic pigs, 2.5 µg of DNA samples were digested overnight with *BamH*I and *Xba*I, separated by electrophoresis on a 0.8% agarose gel, denatured, and transferred to a positively charged nylon membrane (Roche, USA), and then immobilized by UV cross-linking. Southern blot analysis was performed using the 649 bp fragment (described above) labeled with digoxigenin-dUTP as a probe in accordance with the protocol of the DIG High Prime DNA Labeling and Detection Starter Kit II (Roche, USA).

## Supporting Information

Figure S1 **Typical standard curve plot for calculation of lentiviral particle numbers with quantitative RT-PCR using serial dilution of lentivirus.**
(TIF)

Figure S2 **Detection of the transgene in pig ear tissue by PCR in piglets.** A: 3 days old (n = 6), B: 60 days old (n = 6), C: 120 days old (n = 6), D: 180 days old (n = 6), or E: 270 days old (n = 4, one piglet died and one was sacrificed). Lane M: DNA Ladder DL2000 (2000, 1000, 750, 500, 250 and 100 bp from top to bottom) in each figure. Lanes 1 to 6 in A, B, C and D and lanes 3 to 6 in E were resulting PCR products (649 bp in length) from transgenic pig ear DNA. Lanes 7 in A, 8 and 9 in B, 8 in C, 7 in D and 2 in E were negative controls with normal pig ear DNA as template; and lanes 8 in A, 10 in B, 9 in C and 1 in E were no template controls. All negative controls did not produce any specific PCR products.
(TIF)

Figure S3 **PCR amplification of 649 bp fragment from spermatozoa DNA.** Lane M: DNA Ladder DL2000 (2000, 1000, 750, 500, 250 and 100 bp from top to bottom). Lane 1: Negative control replacing template with H_2_O. Lane 2: Positive control with pshRNA-copGFP plasmid as template. Lanes 3–6: Semen samples from 3 male piglets, which were positive for PCR detection in ear DNA, clearly showing here specific PCR products.
(TIF)

Figure S4 **PCR detection of transgene in organs and tissues from one piglet.** Lane M (A and B): DNA Ladder DL2000 (2000, 1000, 750, 500, 250 and 100 bp from top to bottom). Lane A: Normal pig ear DNA control. Lane B: control without any template. Lanes 1–14: PCR amplification of specific DNA from heart, liver, spleen, kidney, lung, stomach, brain, ovary, cerebral cortex, belly fat, semitendinosus muscle, semimembransus muscle, longissimus dorsi muscle and duodenum, respectively.
(TIF)

Figure S5 **RT-PCR detection of transgene expression in organs and tissues from one piglet, and fluorescence imaging in kidney and ovary.** (A) reverse transcription PCR. All samples produced specific products. Lanes 1–10 in A: RT-PCR results of EGFP mRNA from heart, kidney, ovary, duodenum, liver, spleen, stomach, cerebral cortex, belly fat and lung. A 649 bp fragment was amplified in kidney and ovary, and more weakly in heart and lung. Green fluorescence was seen in kidney (B) and ovary (C) as indicated by arrows.
(TIF)

Figure S6 **Imunohistochemical detection of EGFP expressed in ear of PCR-positive transgenic pigs.** Positive (brown) staining was observed in cytoplasm of most ear cells from two pigs. A (200×) was for pig No. 18 and B (200×) was for No. 41. C (200×) was control sample from normal pig ear with negative staining. All nucleus were stained to be blue.
(TIF)

Figure S7 **PCR detection of offspring of one PCR-positive boar.** Lane M: DNA Ladder DL2000 (2000, 1000, 750, 500, 250 and 100 bp from top to bottom). Lane 1–10 are PCR amplification of specific DNA from piglets (piglets 1–5 were from one sow, and 6–10 were from the other.), they all give out positive results. Lane 11, 12 are the negative control with normal pig ear DNA and DNA-free H_2_O, respectively. Lane 13 is the PCR production from positive control.
(TIF)
