# Supplementary figures and images for: Production of Transgenic Pigs Mediated by Pseudotyped Lentivirus and Sperm
Source: PLoS One. 2012 Apr 20;7(4):e35335. doi: 10.1371/journal.pone.0035335 (PMC3335058; doi:10.1371/journal.pone.0035335)

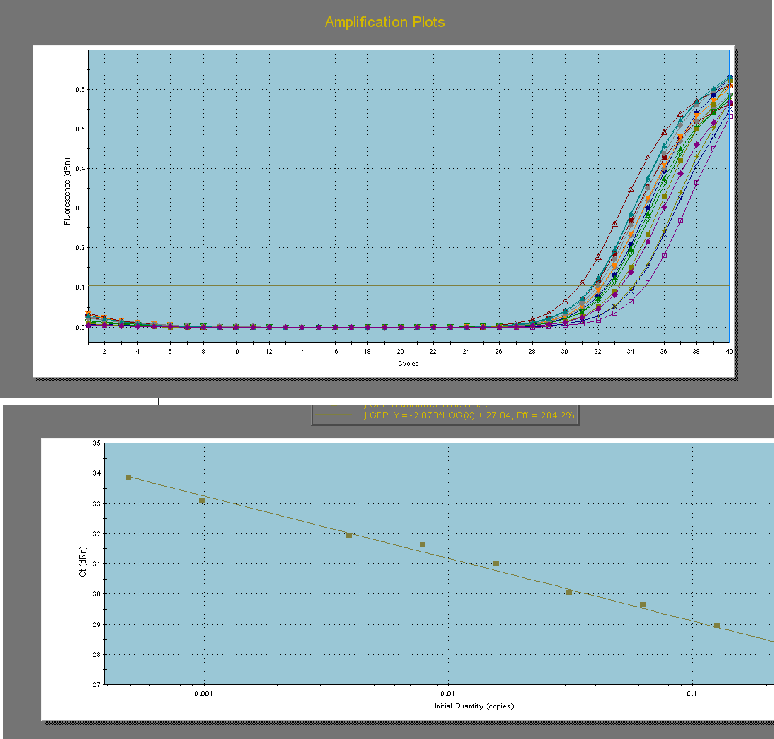

Supplement: Figure S1 — Typical standard curve plot for calculation of lentiviral particle numbers with quantitative RT-PCR using serial dilution of lentivirus. (TIF) [file pone.0035335.s001.tif]

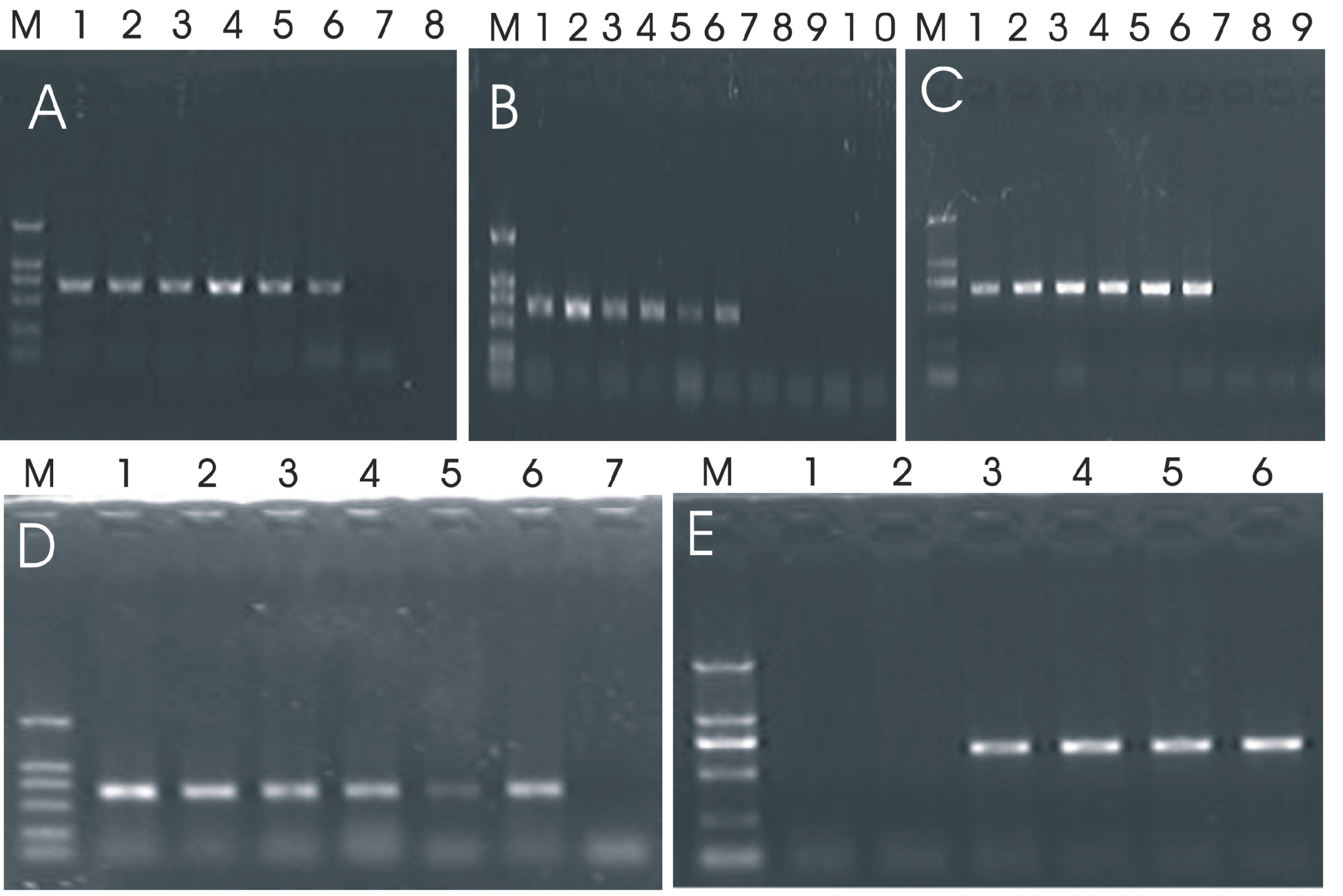

Supplement: Figure S2 — Detection of the transgene in pig ear tissue by PCR in piglets. A: 3 days old (n = 6), B: 60 days old (n = 6), C: 120 days old (n = 6), D: 180 days old (n = 6), or E: 270 days old (n = 4, one piglet died and one was sacrificed). Lane M: DNA Ladder DL2000 (2000, 1000, 750, 500, 250 and 100 bp from top to bottom) in each figure. Lanes 1 to 6 in A, B, C and D and lanes 3 to 6 in E were resulting PCR products (649 bp in length) from transgenic pig ear DNA. Lanes 7 in A, 8 and 9 in B, 8 in C, 7 in D and 2 in E were negative controls with normal pig ear DNA as template; and lanes 8 in A, 10 in B, 9 in C and 1 in E were no template controls. All negative controls did not produce any specific PCR products. (TIF) [file pone.0035335.s002.tif]

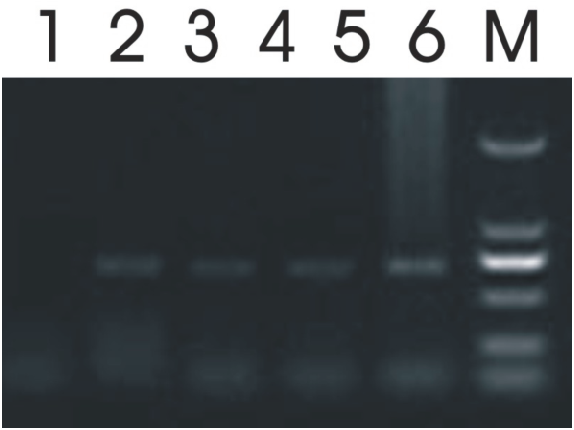

Supplement: Figure S3 — PCR amplification of 649 bp fragment from spermatozoa DNA. Lane M: DNA Ladder DL2000 (2000, 1000, 750, 500, 250 and 100 bp from top to bottom). Lane 1: Negative control replacing template with H2O. Lane 2: Positive control with pshRNA-copGFP plasmid as template. Lanes 3–6: Semen samples from 3 male piglets, which were positive for PCR detection in ear DNA, clearly showing here specific PCR products. (TIF) [file pone.0035335.s003.tif]

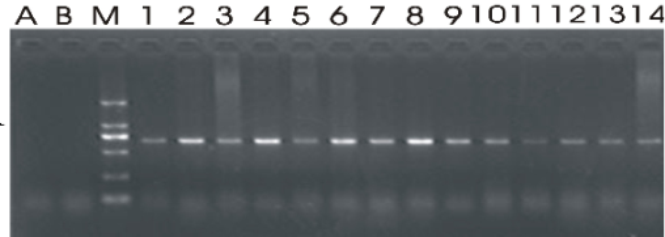

Supplement: Figure S4 — PCR detection of transgene in organs and tissues from one piglet. Lane M (A and B): DNA Ladder DL2000 (2000, 1000, 750, 500, 250 and 100 bp from top to bottom). Lane A: Normal pig ear DNA control. Lane B: control without any template. Lanes 1–14: PCR amplification of specific DNA from heart, liver, spleen, kidney, lung, stomach, brain, ovary, cerebral cortex, belly fat, semitendinosus muscle, semimembransus muscle, longissimus dorsi muscle and duodenum, respectively. (TIF) [file pone.0035335.s004.tif]

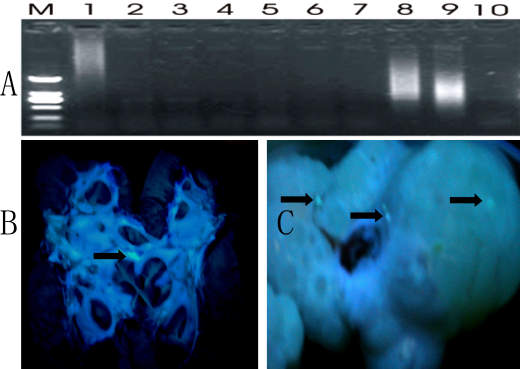

Supplement: Figure S5 — RT-PCR detection of transgene expression in organs and tissues from one piglet, and fluorescence imaging in kidney and ovary. (A) reverse transcription PCR. All samples produced specific products. Lanes 1–10 in A: RT-PCR results of EGFP mRNA from heart, kidney, ovary, duodenum, liver, spleen, stomach, cerebral cortex, belly fat and lung. A 649 bp fragment was amplified in kidney and ovary, and more weakly in heart and lung. Green fluorescence was seen in kidney (B) and ovary (C) as indicated by arrows. (TIF) [file pone.0035335.s005.tif]

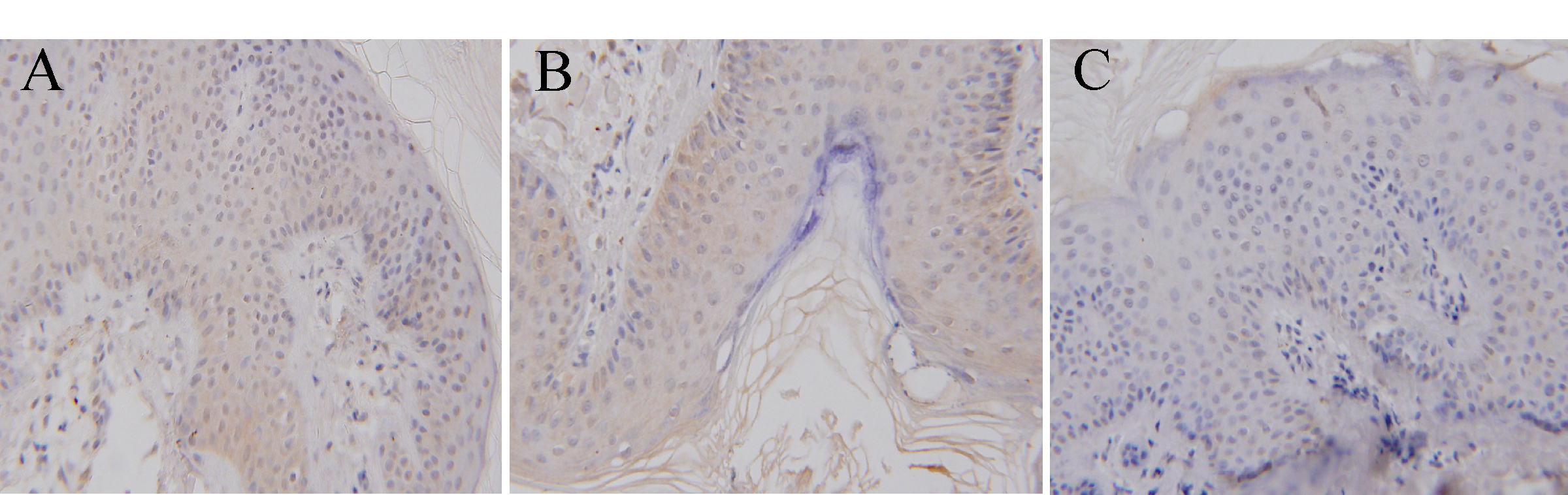

Supplement: Figure S6 — Imunohistochemical detection of EGFP expressed in ear of PCR-positive transgenic pigs. Positive (brown) staining was observed in cytoplasm of most ear cells from two pigs. A (200×) was for pig No. 18 and B (200×) was for No. 41. C (200×) was control sample from normal pig ear with negative staining. All nucleus were stained to be blue. (TIF) [file pone.0035335.s006.tif]

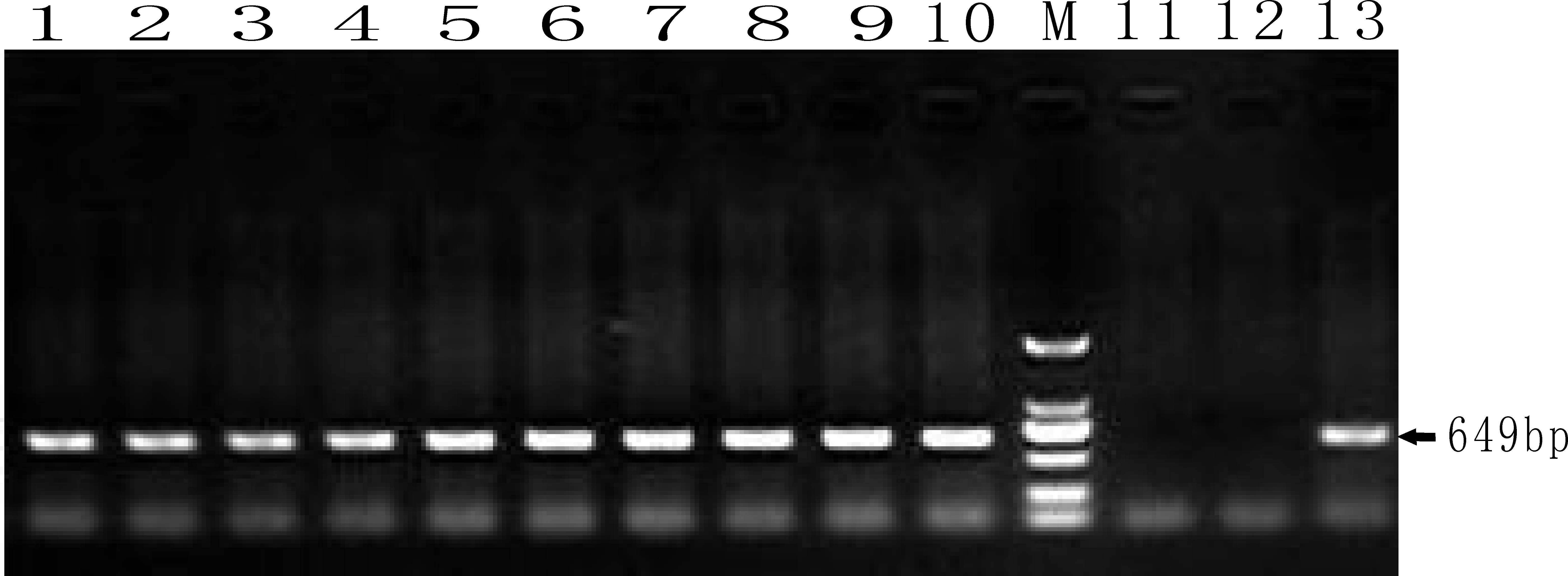

Supplement: Figure S7 — PCR detection of offspring of one PCR-positive boar. Lane M: DNA Ladder DL2000 (2000, 1000, 750, 500, 250 and 100 bp from top to bottom). Lane 1–10 are PCR amplification of specific DNA from piglets (piglets 1–5 were from one sow, and 6–10 were from the other.), they all give out positive results. Lane 11, 12 are the negative control with normal pig ear DNA and DNA-free H2O, respectively. Lane 13 is the PCR production from positive control. (TIF) [file pone.0035335.s007.tif]
